# Association Between Periodontal Pathogens and Inflammation in Patients with Acute Coronary Syndromes

**DOI:** 10.3390/ijms26094360

**Published:** 2025-05-03

**Authors:** Ioana-Patricia Rodean, Vasile-Bogdan Halațiu, Teodora Maria Popa, Emanuel Blîndu, Theofana Mihăilă, Constantin Țolescu, Andrei Modiga, Imre Benedek, Theodora Benedek

**Affiliations:** 1 George Emil Palade University of Medicine, Pharmacy, Science, and Technology of Targu Mures, 540139 Targu Mures, Romania; ioana.rodean@umfst.ro (I.-P.R.); theofana_m@yahoo.com (T.M.); andrei.modiga@umfst.ro (A.M.); imre.benedek@umfst.ro (I.B.); theodora.benedek@umfst.ro (T.B.); 2 Cardiology Department, Emergency Clinical County Hospital of Targu Mures, 540136 Targu Mures, Romania; 3 Center of Advanced Research in Multimodality Cardiac Imaging, CardioMed Medical Center, 540124 Targu Mures, Romania; 4 Emergency Department, Emergency Clinical County Hospital of Targu Mures, 540136 Targu Mures, Romania

**Keywords:** acute coronary syndrome, inflammation, lipid profile, periodontal disease

## Abstract

(1) The link between periodontal disease (PD) and acute coronary syndromes (ACSs) is predominantly attributed to the atherosclerotic process, mediated by systemic inflammation. However, the correlation between the severity of PD, characterized by the presence of periodontal pathogens, and systemic inflammation in patients with ACS remains inadequately clarified. (2) This study aims to assess the association between the severity of PD and systemic inflammatory biomarkers, along with lipid profiles, in patients with ACS. (3) In total, 42 patients with ACS and concomitant PD were divided into two groups based on the presence of periodontal pathogens belonging to the red or red-orange complexes. Group 1–29 patients displayed pathogens from the red complex (RC) and group 2–13 patients displayed pathogens from the red-orange complex (ROC). All participants underwent a comprehensive dental examination, including DNA sampling from the periodontal pockets for pathogen detection. Systemic inflammation was evaluated alongside assessments of lipid profiles. (4) Inflammatory markers were more pronounced in the RC group compared with the ROC group. Moreover, patients in the RC group showed significantly higher monocyte-to-lymphocyte ratios (0.41 ± 0.20 vs. 0.28 ± 0.12; *p* = 0.002), platelet-to-lymphocyte ratios (139.50 ± 33.85 vs. 100.90 ± 8.84; *p* = 0.02), serum C-reactive protein levels (9.34 ± 1.08 mg/L vs. 5.46 ± 1.03 mg/L; *p* = 0.03), and serum uric acid levels (6.9 ± 0.49 mg/dL vs. 5.4 ± 0.26 mg/dL; *p* = 0.006). Concerning lipid profiles, the RC group exhibited significantly higher low-density lipoprotein cholesterol (LDL) levels (169.60 ± 12.63 mg/dL vs. 106.70 ± 9.34 mg/dL; *p* = 0.0007) and significantly lower high-density lipoprotein cholesterol (HDL) levels (29.29 ± 3.50 mg/dL vs. 39.56 ± 2.07 mg/dL; *p* = 0.002). (5) The severity of PD, indicated by the concomitant presence of pathogens from the red and orange complexes, is associated with an unfavorable lipid profile and elevated inflammatory biomarkers. These findings highlight the potential importance of periodontal intervention in the prevention of ACS.

## 1. Introduction

Periodontal disease (PD) is a multifactorial chronic inflammatory condition resulting from an imbalance in the normal microbiota, which leads to the formation of a biofilm referred to as dental plaque, involving various bacteria, particularly Gram-negative species such as *Porphyromonas gingivalis*, *Prevotella intermedia*, *Fusobacterium nucleatum*, among others. This imbalance contributes to the degradation of the soft and hard tissues supporting the teeth due to oral microbial plaque (in the form of a biofilm or in contact with tartar) often in the absence of hygiene and with repeated bacteremias [1,2,3,4]. The chronic inflammatory process triggers a localized and intricate immune response by the host, leading to the progressive destruction of the periodontal supporting tissues and, if left untreated, potentially culminating in tooth loss [5,6,7,8].

Over 700 bacterial species have been identified in the oral microbiome, some of which, under specific conditions, may exhibit abnormal expression, contributing to the onset of PD [9,10]. However, advances in whole genome deep sequencing have shown the existence of about five times more bacterial species, many of which have yet to be described, emphasizing the oral microbiome’s complexity and diversity. Periodontal pathogens can act independently or synergistically, amplifying their effects through co-infection. Regardless of the underlying mechanism, this interaction ultimately leads to the development of a chronic inflammation status, initially localized to the gingiva and subsequently progressing to a systemic condition [11,12]. In contrast, the oral microbiome can experience substantial changes over a short period, both in its composition and activity.

Among periodontal pathogenic bacteria, the most frequently studied species include *Porphyromonas gingivalis*, *Tannerella forsythia*, *Treponema denticola*, *Prevotella intermedia,* and *Aggregatibacter actinomycetemcomitans*. These microorganisms are considered to possess high virulence factors, which play a crucial role in neutralizing the host’s defense mechanisms and contributing to the destruction of periodontal tissue [13]. The red complex (RC) comprises highly aggressive microorganisms implicated in the etiopathogenesis of PD. These bacteria are strongly associated with PD. In contrast, the orange complex (OC) consists of microorganisms with lower virulence compared with those in the RC. While typically insufficient to cause severe forms of PD on their own, these bacteria enhance the pathogenic activity of RC species, thereby contributing to heightened virulence [14,15]. Recent advancements in diagnostic techniques have integrated methods aiming to identify bacterial DNA, RNA, proteins, and metabolites. Among the most employed diagnostic approaches are bacterial cultures, microscopy, gel-based methods, polymerase chain reaction (PCR), bacterial RNA sequencing, and next-generation sequencing (NGS). The NGS, also known as high-throughput sequencing, allows for the simultaneous sequencing of hundreds of genes, providing a deeper coverage of the microbial community [16,17].

C-reactive protein (CRP) is the most significant inflammatory marker involved in both cardiovascular and periodontal diseases. Evidence from the literature indicates that the human gingiva, particularly the periodontium, represents the key site for the synthesis of CRP [3,18]. The activity of pro-inflammatory cytokines induces the degradation of gingival structures, resulting in an elevated secretion of CRP. Furthermore, elevated CRP levels are directly associated with an intensified inflammatory response, thereby reflecting the severity of PD [18]. The potential association between ACS and PD is hypothesized to involve inflammatory cascade, both locally (gingival site) and at the systemic level. Moreover, it has been observed that this pro-inflammatory state correlates directly with the severity of PD. Thus, the more severe the PD, the higher the risk of developing ACS [18,19,20,21,22]. Data in the literature indicate a potential association between specific periodontal pathogens and acute coronary syndrome. Organisms such as *Porphyromonas gingivalis, Aggregatibacter actinomycetemcomitans*, and *Prevotella intermedia* are involved in systemic inflammatory processes that may exacerbate atherosclerotic lesions, thereby elevating cardiovascular risk [23].

Thus, PD is a complex multifactorial inflammatory condition that plays a key role in the atherosclerotic process through both a direct mechanism, such as the activity of periodontal pathogenic microorganisms, and an indirect one, by triggering ACS. However, the precise link between PD and ACS remains insufficiently understood. This study aims to investigate the correlation between periodontal pathogens and the onset and progression of ACS.

## 2. Results

### 2.1. Basic Characteristics of the Study Population

The study cohort comprised 42 patients diagnosed with acute coronary syndrome (ACS) and concomitant PD. Participants were divided into two groups based on the presence of periodontal pathogens from the red complex (RC) or red-orange complex (ROC) which defined the severity of the PD. Group 1 included 29 patients with pathogens from the RC and exhibiting a more severe form of PD, while Group 2 consisted of 13 patients with periodontal infections involving pathogens from the ROC and presenting with a less severe form of PD.

Table 1 presents the demographic and clinical characteristics of the patients. With respect to the distribution of pathogenic bacteria in the study population, it was observed that microorganisms belonging to the RC predominated, including: *Porphyromonas gingivalis*, *Tannerella forsythia,* and *Treponema denticola* (*p* = 0.0009).

### 2.2. Severity of the Periodontal Disease, Inflammatory Status, and Cardiovascular Outcomes

**Figure 1:**
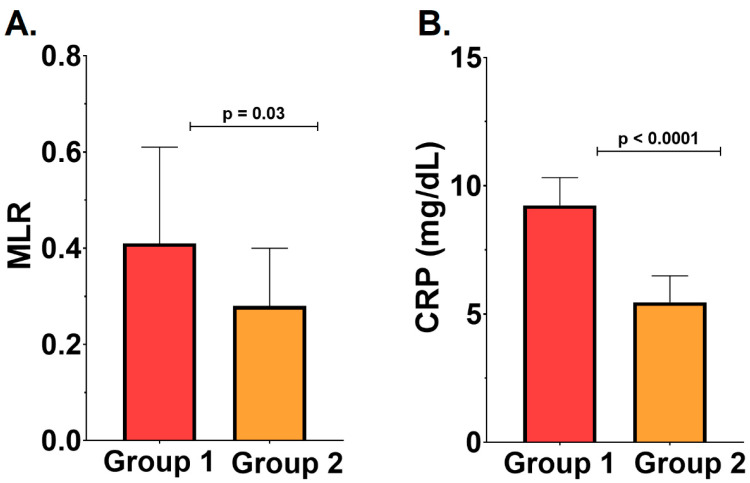
(**A**). Monocyte-to-lymphocyte ratio (MLR) in group 1 versus group 2. (**B**). C-reactive protein (CRP) in group 1 versus group 2. All results are expressed as mean ± standard deviation.

Inflammation was more pronounced in the RC group compared with the ROC group. Patients in the RC group exhibited significantly higher values for the monocyte-to-lymphocyte ratio (MLR) (0.41 ± 0.20 vs. 0.28 ± 0.12, *p* = 0.002) and CRP serum levels (9.24 ± 1.08 mg/L vs. 5.46 ± 1.03 mg/L, *p* = 0.03) compared with the ROC group—Figure 1. No significant difference was noted between the two groups regarding the neutrophil-to-lymphocyte ratio (*p* > 0.05).

**Figure 2:**
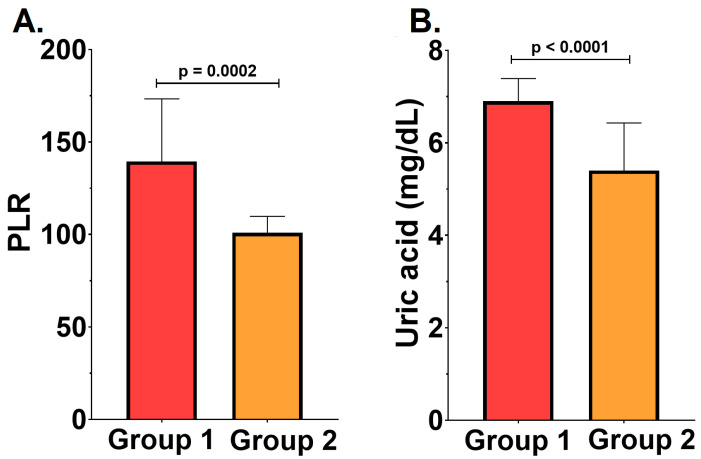
(**A**)**.** Platelet-to-lymphocyte ratio (PLR) in group 1 versus group 2. (**B**). Serum uric acid in group 1 versus group 2. All results are expressed as mean ± standard deviation.

In addition, the platelet-to-lymphocyte ratio (PLR) and serum uric acid levels were evaluated as markers for estimating long-term mortality and prognostic outcomes. Significantly higher PLR (139.5 ± 33.85 vs. 100.90 ± 8.84, *p* = 0.02) and uric acid levels (6.9 ± 0.49 mg/dL vs. 5.4 ± 1.03 mg/dL, *p* = 0.03) were identified in the RC group—Figure 2. These results suggest that the severity of PD correlates with increased mortality and a more unfavorable outcome.

### 2.3. Severity of the Periodontal Disease and the Lipid Profile

**Figure 3:**
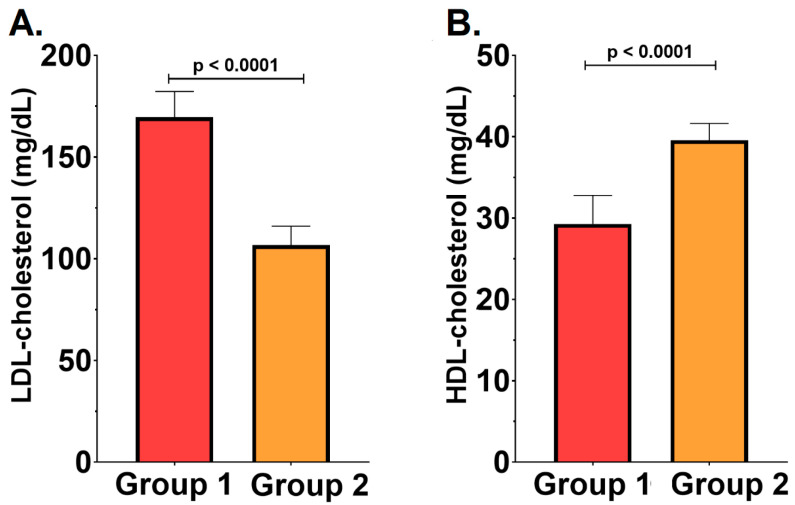
(**A**). Low-density lipoprotein cholesterol in group 1 vs. group 2. (**B**). High-density lipoprotein cholesterol in group 1 vs. group 2. All results are expressed as mean ± standard deviation.

To assess the lipid profile and the association between periodontal pathogens and cardiovascular risk, total cholesterol, low-density lipoprotein (LDL), high-density lipoprotein (HDL) fractions, and tryglicerides (TG) levels were analyzed. It was observed that patients with severe PD, belonging to the RC group, demonstrated a poorer lipid profile, with significantly elevated LDL cholesterol levels (169.60 ± 12.63 mg/dL vs. 106.70 ± 9.34 mg/dL, *p* = 0.0007) and decreased HDL cholesterol (29.29 ± 3.50 mg/dL vs. 39.56 ± 2.07 mg/dL, *p* = 0.002), compared with those with milder forms of disease—Figure 3. However, no significant differences were observed regarding total cholesterol or TG levels between the groups (both *p* > 0.05).

## 3. Discussion

Inflammation, together with the immune response of the host, represents the central mechanism underlying the pathogenesis of atherosclerosis. Nonetheless, several risk factors have been identified as key contributors to the initiation and progression of atherosclerotic lesions. In parallel, it is well established that PD is a chronic inflammatory condition primarily driven by dysbiosis of the oral microbiome, leading, in its advanced stages, to the destruction of the periodontal tissues and subsequent tooth loss. Recent studies have emphasized that the microbial biofilms associated with PD are predominantly Gram-negative, anaerobic bacterial species [24].

According to the evidence in the literature, age and gender are significant non-modifiable risk factors implicated in the etiopathogenesis of both ACS and PD. Based on this hypothesis, the study revealed that the onset of ACS in patients with co-existing PD, typically occurs between the ages 57–59 years old, with a higher frequency observed in females, particularly those with obesity. However, severe forms of PD were more commonly associated with male patients and with a positive family history of CVD.

Comparable results were reported by Persson et al., who investigated ACS co-existing with PD after the age of 60 years [25]. Furthermore, the study by Gita et al. indicated that the concurrent presence of these two conditions typically occurs around the age of 58.24 ± 1.27 years, with a higher prevalence in males compared with females [26]. However, the study conducted by Nordendhal revealed that the simultaneous occurrence of ACS and PD, especially in its severe forms, is three times more common in females and individuals under 65 years old [27].

Analyzing the presence of comorbidities and/or manifest atherosclerosis (e.g., stroke, previous MI, PAD), it was observed that most patients were hypertensive and with CHF. Furthermore, it was proved that only a small percentage of the enrolled subjects were smokers, presented with a history of cardiovascular events (MI/stroke), had DM, or suffered from CKD. Data from the literature reveal a slight predominance of obesity, hypertension, and dyslipidemia in patients with PD and ACS [28]. Similar results were reported in different studies, which demonstrated that among patients who presented acute vascular events, no significant differences were obtained between those with concomitant PD and the control group regarding smoking status, hypertension, dyslipidemia, DM, or BMI [29]. In contrast, a study conducted by Lahdentausta et al. found a statistically significant association between PD and advanced age, DM, active smoking status, and dyslipidemia [30].

The severity of PD is expressed through the presence of pathogens belonging to the red or orange complexes. Specialized data from the literature have demonstrated that the identification of these pathogens, particularly those from the RC, is associated with advanced forms of PD [31]. Renvert et al. demonstrated that in patients with ACS, pathogens predominantly belong to the RC, as well as pathogens from the orange and yellow complex, proving that in patients with ACS, the bacterial load is up to twice as high compared with those without ACS (*p* < 0.01) [32].

Inflammation, a key factor in the pathogenesis of both PD and ACS, is more pronounced with increasing severity of PD. Also, it was identified in numerous studies that inflammation is a potential predictor of CVD risk. Markers such as procalcitonin, high-sensitivity CRP, and the MLR have been employed as indicators of CHD risk. However, there is a lack of sufficient studies specifically exploring the role of inflammation in predicting CHD risk in patients with PD [33]. In this regard, serum CRP levels were significantly elevated in patients with advanced PD, characterized by the presence of pathogens from the RC. Furthermore, other inflammatory markers, such as MLR and PLR, were found to be associated with severe PD. Elevated PLR values also indicated that patients with advanced PD who experienced ACS exhibited a less favorable prognosis, accompanied by an increased risk of mortality. These observations were corroborated by a statistically significant correlation with serum uric acid level and PD severity. These data were enforced by similar data in the literature. Thus, it was observed that the presence of pathogens from RC is associated with more pronounced inflammation, as evidenced by significantly higher serum levels of CRP. According to a study by Kadhim et al., serum levels of CRP were significantly higher in patients with PD compared with those without periodontal involvement (*p* = 0.001). Moreover, CRP level was significantly reduced after the initiation of statin therapy [34]. These findings are further supported by observations indicating that serum levels of CRP are elevated in patients with ACS and concomitant moderate or severe PD (*p* < 0.05) [35].

The novelty of this study stems from the limited data available regarding the link between PD and inflammatory hematological markers, specifically MLR and PLR. In an experimental study conducted on dogs, the PLR value was higher in animals with periodontitis compared with healthy ones (*p* = 0.024), but no significant differences were obtained regarding MLR [36]. Recent data suggest that periodontitis exhibits a statistically significant relationship with NLR and MLR, while its association with PLR remains debatable. These changes can be attributed to the fact that systemic inflammation triggers a significant deterioration in blood cell counts, thereby impacting the modification of the respective indices [37]. Similar data were obtained in a Chinese cohort in patients with CVD, PD, and mental disorders [33]. The NLR was also elevated in patients with PD compared with a healthy population [38]. The results of our study did not demonstrate a significant association between the NLR and the severity of PD in patients with ACS. These findings may be attributed to the chronic inflammatory processes that are characteristic of both conditions, as well as moderate forms of PD, where inflammation may either be highly exacerbated or relatively diminished.

An increasing body of research highlights the critical role of uric acid in the pathogenesis of inflammatory diseases. Uric acid, as an inflammatory mediator, may contribute to vascular endothelial dysfunction and promote smooth muscle cell proliferation, thereby accelerating the progression of atherosclerosis. Additionally, inadequate dental hygiene, such as irregular brushing, may exacerbate the formation of dental calculus, a process potentially influenced by elevated uric acid levels. Thus, hyperuricemia is associated with poor oral hygiene and ACS [39]. A recent study reported that patients with ACS and caries burden exhibit significantly elevated serum levels of CRP and uric acid compared with healthy individuals [40]. Conversely, the study conducted by Chabuk et al. did not identify a statistically significant correlation between serum uric levels and the severity of PD in patients with ACS. Nonetheless, the results showed that the severity of the periodontal disease was associated with greater CRP levels [41]. These findings are further corroborated by another study that establish a clear association between hyperuricemia and PD, especially in patients with ACS [42]. In our study, a significant positive correlation was observed between serum uric acid levels and the severity of PD, as evidenced by the presence of pathogens from the RC. Thus, it is further demonstrated that the inflammatory process represents the key mechanism linking the two conditions, and as the inflammation becomes more exacerbated, the long-term outcomes in these patients are more unfavorable.

Nevertheless, our study highlighted that patients with both PD and ACS exhibit a more disrupted lipid profile, characterized by higher LDL. However, no significant correlation was found with total cholesterol or TG levels. Thus, the cardiovascular risk is higher in the presence of both pathologies, with long-term prognosis being burdened by numerous complications. It is also well established that inflammation underlies all oxidative processes involved in atherosclerosis, including LDL oxidation, which may explain the isolated increase in LDL levels observed in patients with severe PD and ACS. Similar results have been described in the literature, which not only highlight a significant correlation between LDL and the severity of PD, but also between serum levels of TG [43,44,45]. Also, it was observed that cholesterol level is higher in patients with periodontitis [46]. This suggests that poor lipid profile could serve as a risk factor for PD. Consequently, individuals with progressive PD should monitor and manage their LDL and TG levels.

In recent years, there has been a growing interest in the relationship between genetic information, periodontal disease, and overall health. Although our current study lacks direct data on host genotypes, it is critical to note that variations in these genotypes may have a significant impact on dysregulated immune responses associated with periodontitis. This dysregulation affects not only oral health but also systemic conditions, especially in patients with metabolic disorders.

According to Nibali et al. [47,48], genetic factors associated with systemic health can interact with the oral microbiome, implying that periodontal disease may serve as a critical nexus through which inflammation influences conditions such as coronary atherosclerosis. Understanding these interactions is critical for developing personalized therapeutic strategies and assessing cardiovascular risk in individuals with periodontal disease. Furthermore, the investigation of gingival crevicular fluid biomarkers represent a promising area for future research. Data in the literature emphasize the utility of gingival crevicular fluid as a diagnostic tool in patients with metabolic dysfunction. These biomarkers may serve as indicators of the underlying inflammatory processes that link periodontitis and acute coronary syndromes.

The main limitation of this study is the relatively small patient cohort, which may impact the results. Additionally, the statistical differences observed in comparison with existing data in the literature could be attributed to the fact that many studies on periodontal pathogens and their relationship with CVD have been conducted on animal models or have focused on conditions other than ACS. Furthermore, inter-racial differences may significantly contribute to the observed variations. The lack of comprehensive studies on gingival crevicular fluid biomarkers in the context of metabolic dysfunction highlights a significant gap in the present research. Future studies with larger cohorts are warranted to confirm these preliminary results. The absence of a control group may limit the relevance of the bacteriological analysis, underscoring the need for caution in interpreting the findings.

The discrepancies observed between the findings of this study and those reported in the existing literature may arise from the limited number of studies exploring this hypothesis. Consequently, this research is among the first to comprehensively analyze the association between PD severity, as indicated by the presence of periodontal pathogens, and cardiovascular risk in patients with ACS.

It is important to acknowledge that while our study focuses on the potential association between periodontal disease and acute coronary syndromes, the etiology of periodontal disease is often multifactorial. Various systemic conditions and pathological processes can significantly influence the onset, progression, and severity of periodontal disease, independent of or in conjunction with the presence of ACS [49].

## 4. Materials and Methods

### 4.1. Study Population

This study was designed as a case–control observational study conducted within the Cardiology Department of the County Emergency Clinical Hospital in TarguMures, Romania. The primary objective was to investigate the relationship between the severity of PD, determined by the presence of periodontal pathogens, and systemic inflammation, as well as lipid profile alterations in patients diagnosed with ACS. Between January and December 2023, patients with ACS and coexisting PD were enrolled. Comprehensive data, including personal medical history, family history, demographic information (e.g., gender, age body mass index (BMI), smoking status) were collected for each patient. Smoking status was determined based on the patient’s medical history, with patients categorized as smokers if they reported smoking for a minimum of one month prior to study enrollment. All data were recorded anonymously.

The diagnosis of ACS was made in accordance with the most recent ESC Guidelines for the management of acute coronary syndromes [50]. This process included the differentiation between non-ST-segment elevation (NSTEMI) and ST-segment elevation myocardial infarction (STEMI), based on the presence of myocardial ischemia pattern observed on electrocardiograms and the levels of cardiac biomarkers.

The exclusion criteria for this study were as follows: malignancies, life expectancy less than 1 year, pregnancy, acute renal failure, drug-induced gingival hyperplasia, recent treatment with antibiotics, autoimmune and chronic inflammatory diseases.

### 4.2. Serum Biomarkers

Peripheral venous blood samples were collected from the antecubital vein at the time of intensive care unit admission. The samples were then centrifuged at 3000 rotations per minute, and the platelet-poor supernatant was carefully aspirated and transferred into sterile vials. Furthermore, these samples were stored at −80 °C for later analysis. Serum analysis was conducted using the equipment available in the Cardiology Department of the TarguMures County Clinical Emergency Hospital. To assess all biological markers, the following analyzers were used: Dimension EXL 200 analyzer (Siemens Healthineers, Erlangen, Germany) and Cobas Integra Plus analyzer (Roche Diagnostics GmbH, Manheim, Germany).

### 4.3. Cardiovascular Risk Factors

Smoking status was determined based on the patient’s medical history, with patients categorized as smokers if they reported smoking for a minimum of one month prior to study enrollment. Hypertension was defined according to the European Hypertension Guidelines [51] as having a blood pressure reading of 140/90 mmHg or higher, or receiving ongoing antihypertensive treatment. Obesity was defined as a BMI exceeding 30 kg/m^2^. Furthermore, the study documented the presence of a history of diabetes mellitus (DM), atrial fibrillation (AF), chronic heart failure (CHF), chronic kidney disease (CKD), peripheral artery disease (PAD), stroke, and previous myocardial infarction.

### 4.4. Dental Examination

A thorough oral examination was performed by a qualified periodontist to evaluate the periodontal status prior to participant inclusion in the study. The diagnosis was based on the presence of markers indicative of the severity of PD, as well as the participant’s medical history, including prior or ongoing specific medical or surgical treatments for PD.

Microbial samples were obtained from the deepest gingival pocket identified during the periodontal assessment. For each patient, bacterial DNA was isolated from the paper cones using MicroID kits targeting periodontal pathogens from RC and OC (Heine Lifescience, Nehren, Germany), in accordance with the manufacturer’s instructions. Following collection, all samples were promptly transported to Germany for analysis. The results were sent as comprehensive reports detailing the infection type, the specific pathogens identified, and the individualized antibiogram for each patient.

The isolation process involved carefully collecting samples from the paper cones, which served as a medium for bacterial growth and preservation. The MicroID kits utilized a combination of lysis buffers and purification columns that facilitated the extraction of genomic DNA from diverse bacterial species, including those classified within the RC and OC of periodontal pathogens.

Following the manufacturer’s protocols, the paper cones were subjected to mechanical disruption and enzymatic digestion to ensure efficient lysis of the bacterial cells. Once the DNA was released, it was purified, concentrating the target DNA while removing inhibitors that could interfere with downstream applications, such as polymerase chain reaction (PCR) or sequencing analyses. The efficacy of the extraction process was subsequently validated through quantitative and qualitative assessments of the isolated DNA, ensuring its suitability for further molecular characterization of the periodontal microbiome. This approach provided a reliable basis for investigating the pathogenic profiles associated with periodontal disease, thus, contributing to our understanding of microbial ecology in oral health.

### 4.5. Statistical Analysis

Statistical analysis was performed using Graph Pad InStat version 3.10 software (GraphPad Software Inc., San Diego, CA, USA). Normality tests were applied to all data prior to analysis. Categorical variables were reported as absolute numbers and percentages, while continuous variables were expressed as a mean ± standard deviation (SD). Group comparisons were performed using the Mann–Whitney test for non-normally distributed variables, Student’s *t*-test for normally distributed variables, and Fisher’s exact test for categorical data. A significance level (alpha) of 0.05 was applied to all statistical tests.

### 4.6. Ethical Consideration

This study is part of the ATHERODENT trial (NCT03395041) and received ethical approval from the Ethics Committee of the George Emil Palade University of Medicine, Pharmacy, Science, and Technology of Targu Mures (approval no. 351/12 December 2017). Written informed consent was obtained from all participants prior to any study-related procedures. Moreover, all study data as well as therapeutic and diagnostic protocols were managed in compliance with patient confidentiality rights.

## 5. Conclusions

Patients with ACS exhibit a more severe periodontal condition compared with individuals without CVD. The greater the severity of PD, the more pronounced the inflammation, and the prognosis for these patients tends to be less favorable. These findings contribute to a deeper understanding of the role of inflammation as a central mechanism in both PD and ACS. Furthermore, they establish a basis for future studies, highlighting the importance of better lipid profile management to diminish the cardiovascular risk and to improve the outcomes. Additionally, it is crucial to identify all patients with PD, from the early stages, and to conduct more through cardiovascular screening to reduce the risk of acute events.

## Figures and Tables

**Table 1:** Baseline characteristics of the study population.

	Study Population (n = 42)	Group 1—RC (n = 29)	Group 2—ROC (n = 13)	*p* Value
Age (years)	58.34 ± 10.7	59 ± 10.64	57.69 ± 10.76	0.71
BMI (kg/m^2^)	30.41 ± 4.97	30.65 ± 4.995	30.17 ± 4.95	0.78
Male gender (n, %)	20 (48%)	17 (59%)	3 (23%)	**0.04**
DM (n, %)	16 (38%)	13 (45%)	3 (23%)	0.30
AF (n, %)	2 (5%)	1 (3%)	1 (8%)	0.53
CHF (n, %)	28 (97%)	19 (66%)	9 (69%)	0.9
Hypertension (n, %)	37 (88%)	25 (86%)	12 (92%)	0.9
Smoker status (n, %)	13 (31%)	9 (31%)	4 (31%)	0.9
CKD (n, %)	12 (29%)	9 (31%)	3 (23%)	0.72
PAD (n, %)	6 (14%)	3 (10%)	3 (23%)	0.35
Stroke (n, %)	2 (5%)	1 (3%)	1 (8%)	0.53
Previous MI (n, %)	14 (33%)	11 (38%)	3 (23%)	0.49
Family history of CVD (n, %)	22 (52%)	20 (69%)	2 (15%)	**0.002**

The values are expressed as mean ± standard deviation, absolute values, and percentages, respectively. *p*-values refer to between-group comparisons based on unpaired *t*-test. Significant *p*-values are shown in bold. BMI—body mass index; DM—diabetes mellitus; AF—atrial fibrillation; CHF—congestive heart failure; CKD—chronic kidney disease; PAD—peripheral artery disease; MI—myocardial infarction; CVD—cardiovascular disease.

## Data Availability

Archived datasets are available upon request by any interested third party.
